# Acetylsalicylic acid as a potential pediatric health hazard: legislative aspects concerning accidental intoxications in the European Union

**DOI:** 10.1186/s12995-016-0118-5

**Published:** 2016-07-13

**Authors:** Menen E. Mund, Christoph Gyo, Dörthe Brüggmann, David Quarcoo, David A. Groneberg

**Affiliations:** Institute of Occupational Medicine, Social Medicine and Environmental Medicine, Departments of Female Health and Preventive Medicine, Goethe University, Frankfurt am Main, Theodor-Stern-Kai 7, Frankfurt, 60590 Germany; Department of Obstetrics and Gynecology, Keck School of Medicine, University of Southern California, Los Angeles, CA USA

**Keywords:** Acetylsalicylic acid, Analgesics, Poisoning, Intoxication, Child, Pediatrics, Legislation, European Union, Non-pharmacy

## Abstract

Acetylsalicylic acid is a frequently used medication worldwide. It is not used in pediatrics due its association with Reye syndrome. However, in case of pediatric intoxication, children are more fragile to salicylate poisoning because of their reduced ability of buffer the acid stress. Intoxication leads to a decoupling of oxidative phosphorylation and subsequently to a loss in mitochondrial function. Symptoms of poisoning are diverse; eventually they can lead to the death of the patient. Governmental websites of various EU countries were searched for legal information on acetylsalicylic acid availability in pharmacies and non-pharmacy stores. Various EU countries permit prescription-free sales of acetylsalicylic acid in pharmacies and non-pharmacy stores. In Sweden acetylsalicylic acid 500 mg may be sold in a maximum package size of 20 tablets or effervescent tablets in a non-pharmacy. In the UK a maximum of 16 tablets of acetylsalicylic acid 325 mg is allowed to sell in non-pharmacies. In Ireland acetylsalicylic acid is classified as S2 medication. Subsequently, acetylsalicylic acid is allowed to be sold prescription-free in pharmacies and non-pharmacy stores. In the Netherlands acetylsalicylic acid may only be sold in drug stores or pharmacies. A maximum of 24 tablets of 500 mg is allowed to purchase in a drug store. Several countries in the European Union are permitted to offer acetylsalicylic acid prescription-free in pharmacies and non-pharmacy stores without legal guidance on the storage position within the store. Further research is needed to investigate whether acetylsalicylic acid is located directly accessible to young children within the stores in EU countries which permit prescription-free sales of acetylsalicylic acid.

## Background

**Fig. 1:**
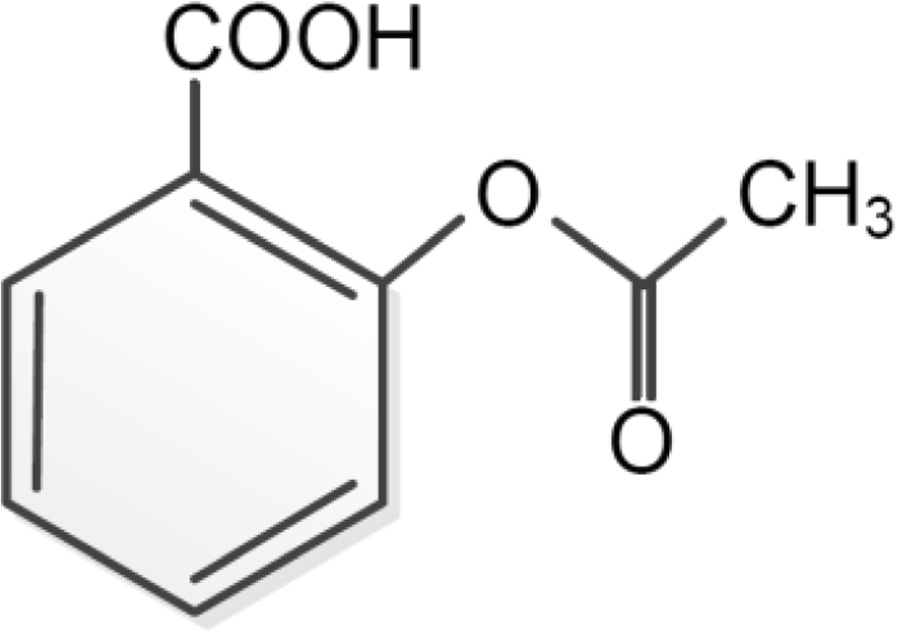
Chemical structure of aspirin, modified after [36]

Recently we published a narrative review about paracetamol as a toxic substance for children [1]. Accordingly, the present review will focus on another important analgesic as a potential pediatric health hazard: acetylsalicylic acid. It is one of the most wildly used analgesic and antiplatelet medication throughout the world. It is commonly known as aspirin, which is the product name introduced by the pharmaceutical company *Bayer AG*. Figure 1 shows the chemical structure of acetylsalicylic acid. In order to avoid Reye syndrome, it is not commonly used in pediatric treatment any more. Nonetheless, acute poisoning in children can occur by accidental ingestion of adult medication [2, 3]. This narrative review summarizes the effects of acetylsalicylic acid intoxication, especially in children. Additionally, it outlines the current legal requirements on acetylsalicylic acid sales in various countries ofthe European Union. The goal was to establish whether the pharmaceutical might be accessible to young children within pharmacies and non-pharmacy stores.

### Acetylsalicylic acid toxicity

The acetylsalicylic acid metabolite salicylate affects most organ systems. It decouples oxidative phosphorylations like the *Krebs cycle* and amino acid synthesis. On a molecular level it increases oxidative stress and subsequently results in a loss of mitochondrial potential; the consequence is a damaged mitochondrial respiratory function. Initially, acetylsalicylic acid poisoning leads to pure respiratory alkalosis due to activation of the respiratory center in the medulla oblongata. Thereafter, acetylsalicylic acid poisoning causes anaerobic oxidation which results in anion gap metabolic acidosis; the undetected anions include salicylates and lactate [4–6]. The typical triad of acetylsalicylic acid intoxication consists of hyperventilation, tinnitus and gastrointestinal symptoms such as vomiting and nausea. Other early symptoms include hematemesis, deafness, lethargy and confusion. Pulmonary edema, hemorrhages and hyperglycemia have also been observed. Serious poisoning can additionally lead to hepatotoxicity. After severe intoxication, central nervous system (CNS) changes like agitation and confusion might occur with a risk of cerebral edema; these symptoms can lead to coma. Acetylsalicylic acid intoxication can eventually result in the death of the patient [2, 7, 8]. One study reported a case of a child with detected high acetylsalicylic acid blood levels suffering from acute myocarditis. These findings suggest a causal relationship between myocarditis and acetylsalicylic acid overdose [9].

### Acetylsalicylic acid intoxication in children

The acetylsalicylic acid metabolite salicylate has been listed amongst the nine pediatric poisons which lead to death in children at low doses [10]. Young children are more fragile to salicylate poisoning because they cannot compensate acid stress as effectively as adults; CNS changes like agitation and restlessness are especially widespread in children [2]. The acute toxic acetylsalicylic acid dosage is considered to be more than 150 mg/kg body weight [8]. Salicylate intoxication is probably under-represented in poison center data because the symptoms are unspecific and intoxication is often not diagnosed as such [7]. A US study showed that unintentional acetylsalicylic acid poisoning in children was a common problem around 1970. At that time it was the most frequent medicine leading to accidental poisoning in children. Improving child-proof pharmaceutical containers lead to a decrease of acetylsalicylic acid intoxication in children under 5 years [11]. Statistical data on acetylsalicylic acid intoxication in children is unfortunately limited. This particular intoxication is often not registered specifically in central statistical databases. Governmental agencies in various countries published reports on child injury. Data is usually based on hospital statistics and death cause statistics; these statistics inform about diverse child injury mechanisms. Poisoning is generally registered as one of them, but the reports do not provide information about particular drugs. Accordingly, they do not present statistical information about drug poisonings with acetylsalicylic acid in children [12–14]. In all four countries which were included in this study poison control centers are installed; they generally function as advisor for telephone enquiries made by medical professionals or the general public. Poison control centers usually document information about calls, for example the reason for the phone call or whether the patient is a child. However, in these reports poisoning incidences are often registered by substance group, for example analgesics, but do not show data about acetylsalicylic acid specifically [15–18].

### Treatment of acute acetylsalicylic acid intoxication

An antidote for acetylsalicylic acid intoxication is not known. Active charcoal should be administered immediately after ingestion to reduce the quantity of absorbed substance. Emesis should not be forced in children suffering from acute acetylsalicylic acid poisoning; whole bowel irrigation or gastric lavage is also not recommended any more [7, 8]. Serum acetylsalicylic acid levels should be identified and blood gas analysis should be performed repeatedly. Severity of intoxication does often not correlate with the absolute serum level; therefore blood analysis should be repeated and used to monitor serum level changes. A nomogram was developed in 1960 by *A.K. Done* to establish a treatment threshold on basis of serum acetylsalicylic acid levels. However, due to serious limitations of the nomogram is not generally used [7]. Further conservative treatments of acetylsalicylic acid poisoning include rehydration and correction of electrolyte aberrations. Urinary alkalinization is important in order to accelerate the ionization of the drug and therefore force renal elimination [19]. Hemodialysis is a treatment which is commonly used in patients after acetylsalicylic acid poisoning, even though no clear regime exists concerning duration and best method. If a decision on hemodialysis is made, alkalinization of urine should nevertheless be promptly administered [20, 21]. Mechanical ventilation should not be applied to patients with acetylsalicylic acid intoxication as it results in abolishment of respiratory alkalosis. Mechanical ventilation aggravates the neurological toxic effects because more salicylate can pass to the CNS [22]. It is suggested that balanced glutathione homeostasis in hepatic cells reduces the cytotoxic effects of acetylsalicylic acid [6].

### Pharmaceutical legislation in European Union countries

European Union countries receive legislation from two different legislative authorities. On the one hand, national governments enact laws which apply to every distinct country individually. On the other hand, laws in EU countries can be enacted by the EU; the European Commission (EC), the European Parliament (EP) and the European Council form the EU legislative body. The EC proposes legislation which is discussed and eventually enacted by the EP and the European Council. Two different types of legislation are performed by the EU: regulations and directives. An EU regulation is instantly enforced as law in all EU member countries at the same time; it excels national law. EU directives are guidelines with a defined goal and time period in which it has to be incorporated into national law. Legislation processes on EU level are complicated; occasionally it takes a long time until a regulation or directive becomes enforced [23–25]. The EU pharmaceutical law consists of diverse regulations and directives concerning numerous pharmaceutical subjects like pharmacovigilance, falsified medical products, drug marketing or regulatory processes. Accessibility of acetylsalicylic acid however is not regulated by European law but by national law [26]. Information about legal requirements for pharmaceutical sales in EU countries was not found in a systematic search in PubMed database. Therefore, five countries of the EU were pre-selected. They were chosen to investigate a cultural variety within the EU: the UK, Ireland, Sweden, the Netherlands and Spain. Governmental websites were used to gain information. Due to different native languages on governmental websites, no single search term could be used during the search. Spain does only allow pharmacy sales of acetylsalicylic acid and was therefore excluded from the study.

### Legislation in Sweden

The *Swedish Medicines Act* enables the *Medical Product Agency* to control and monitor medical items in Sweden. The agency is furthermore entitled to classify drugs into prescription-free and prescription-only medication [27, 28]. The maximum prescription-free package size of acetylsalicylic acid consists of 500 mg per unit with 20 tablets (tab) and 20 effervescent tablets (TEF) per package and in a non-pharmacy store. In a pharmacy, it is legal to purchase 50 tablets or 60 TEF per package [29] (Table1).

**Table 1:** Aspirin availability in Sweden, modified after [29, 37, 38]

Form	Strength	Pack size non-pharmacy	Pack size pharmacy
Tab	250 mg	50 units	50 units
Tab	500 mg	20 units	50 units
TEF	500 mg	20 units	60 units

### Legislation in the United Kingdom

The *Medicines and Healthcare Products Regulatory Agency* is an administrative body of the British *Department of Health*; this agency is responsible for admission, monitoring and safety of pharmaceuticals in the United Kingdom. The *Medicines Act* from 1968 constitutes the legislation of pharmaceuticals. Medicines are divided into three different categories [30, 31]:

- Prescription-only medication
- Pharmacy sales medication
- General sales list medication (GSL)

Certain requirements in package size and strength apply for acetylsalicylic acid in order to be listed as GSL (Table 2). A maximum of 16 tablets of acetylsalicylic acid 325 mg may be offered in a non-pharmacy store [31].

**Table 2:** Requirements for aspirin to listed GSL in the UK, modified after [31]

Form	Strength	Pack size
TEF	500 mg	20 units
Powders or granules	650 mg	10 units
Tab or capsules	325 mg	16 units

### Legislation in Ireland

The *Irish Medicines Board* forms the official agency of the *Department of Health and Children.* This agency regulates pharmaceutical matters like drug safety, drug risks and monitoring. The *Medical Product Regulations Law* regulates prescription and supply of pharmaceuticals. Three categories for medication exist in Ireland. Pharmaceuticals are either classified as:

- General sales medication
- Prescription-controlled medication from *schedule 1* (S1)
- Exemption from S1 medication.

These exemptions are either pharmaceuticals listed in *schedule 2* (S2) or S1 pharmaceuticals which are labeled with maximum dose, maximum daily dose or maximum treatment period. S2 medication can be offered in non-pharmacies [32]. Acetylsalicylic acid is classified as S2 medication and it is labeled with a maximum daily dose of 4.0 g for patients aged more than 12 years old. Subsequently, acetylsalicylic acid is allowed to be sold prescription-free in pharmacies and non-pharmacy stores [32, 33].

### Legislation in the Netherlands

The Dutch medicine law came into effect in 2007 and was enforced by the *Ministry of National Health, Wellbeing and Sports*. This ministry instructs the administrative body *Medicines Evaluation Board* to control pharmaceutical safety and quality. Medications are divided into four different categories in the Netherlands [34]:

- Prescription-only medication (UR)
- Pharmacy-only medication (UA)
- Pharmacy or drug store-only (UAD)
- Open sales medication (AV)

Acetylsalicylic acid does not belong to the AV list; it is not permitted to sell acetylsalicylic acid outside of drug stores or pharmacies. The medication holds UAD status for a maximum pack size of 24 tablets of 500 mg; more than 24 tablets per package may only be sold in pharmacies [35] (Table 3).

**Table 3:** Aspirin availability in the Netherlands, modified after [35]

Form	Strength	Pack size	Status
Tab	500 mg	24 tab	UAD
Tab	500 mg	> 24 tab	UA

## Conclusions

Acetylsalicylic acid is a drug which is not commonly used in pediatrics. However, in case of accidental poisoning, it can lead to severe symptoms. Several countries in the European Union are permitted to offer acetylsalicylic acid prescription-free in pharmacies and non-pharmacy stores without legal guidance on the storage position within the store. Further investigation is needed to analyze whether the pharmaceutical is placed in direct accessibility to young children within these stores.

## Abbreviations

AV, Open sale medication *(algehele verkoop geneesmiddel)*; CNS, Central nervous system; EC, European Commission; EP, European Parliament; GSL, General sales list; S1, Schedule 1; S2, Schedule 2; Tab, tablets; TEF, Effervescent tablets; UA, Pharmacy only medication *(uitsluitend apotheek geneesmiddel)*; UAD, Pharmacy or drug store only *(uitsluitend apotheek of drogist geneesmiddel)*; UR, Prescription only medication *(uitsluitend recept geneesmiddel)*
